# Promotion and consumption of breastmilk substitutes and infant foods in Cambodia, Nepal, Senegal and Tanzania

**DOI:** 10.1111/mcn.12308

**Published:** 2016-04-15

**Authors:** Elizabeth Zehner

**Affiliations:** ^1^ Helen Keller International Washington District of Columbia USA

The World Health Organization (WHO) and UNICEF define optimal infant and young child feeding as practising exclusive breastfeeding from birth through the first 6 months of life and feeding with safe and appropriate complementary foods starting from 6 months of age together with continued breastfeeding for up to 2 years and beyond (WHO & UNICEF 2003). Meeting the nutritional requirements of children from the age of 6 months is challenging when foods fed to children are low in essential micronutrients, low in high quality fats, high in factors that inhibit absorption of nutrients and not adequately dense in calories. To meet the specific nutrient requirements of this vulnerable group, WHO guidelines (Pan American Health Organization & WHO 2003) recommend the use of low‐cost fortified products as needed, along with continued breastfeeding; however, these products need to be promoted in a way that protects both breastfeeding and the consumption of high‐quality local foods.

The International Code of Marketing of Breast‐milk Substitutes (WHO 1981) was adopted by the World Health Assembly (WHA) in 1981 to stop the promotion of breastmilk substitutes, which has been shown to be detrimental to breastfeeding practices. In May 2010, the 63rd World Health Assembly recognized that the promotion of some commercial foods for infants and young children also undermines progress in optimal infant and young child feeding (WHA 2010), and in May 2012, the Assembly requested the director general to provide clarification and guidance on the issue of inappropriate promotion of foods for infants and young children (WHA 2012).

Information on promotion and consumption of foods for infants and young children in countries around the world is limited. Policy makers at both the global and national levels seeking to improve infant feeding practices and specifically those tasked with providing guidance on promotion of commercially produced complementary foods could benefit from more detailed information on the rates of consumption of foods by infants and young children as well as the prevalence and nature of the promotion of these foods.

In response to the call in 2012 by global policy makers for guidance, Helen Keller International, through the Assessment and Research on Child Feeding (ARCH) Project, has conducted research on the promotion of commercially produced foods and their consumption by infants and young children in the largest urban areas of four countries: Cambodia, Nepal, Senegal and Tanzania. The ARCH Project also gathered data on the labelling of commercially produced foods consumed by infants and young children in these four sites. This supplement describes the results from this research.

The four study sites are not only geographically diverse but also vary in legislation governing the promotion of infant foods. Strong laws governing the promotion of breastmilk substitutes and commercially produced complementary foods exist in Nepal and Tanzania, covering products for children up to 12 months and 5 years of age, respectively. Promotion is less strictly regulated in Cambodia (where promotions are permitted with government approval) and in Senegal (where promotion is only prohibited within health facilities).

The first six articles in this supplement describe information collected from mothers of children under the age of 2 years on exposure to promotional practices for breastmilk substitutes, commercially produced complementary foods and commercially produced snack food products. These health facility‐based, cross‐sectional surveys collected information about health system practices related to infant and young child feeding, as well as information on promotion outside health facilities. The surveys also gathered information on infant and young child feeding practices, including consumption of breastmilk substitutes, complementary foods and other commercially produced foods, including snack food products.

In Kathmandu Valley, Nepal, Pries *et al*. report a high prevalence of prelacteal feeding of breastmilk substitutes, with over half of mothers reporting this practice. Reported recommendations from health professionals to use breastmilk substitutes were also prevalent, and mothers who received a recommendation to use a breastmilk substitute from a health worker were more likely to provide a prelacteal feed of a breastmilk substitute as compared with mothers who did not receive a recommendation (Pries *et al.* 2016a).

In the second article from Kathmandu Valley, the authors report that commercially produced snack food products are frequently consumed by young children. While approximately one quarter of children 6–23 months of age had reportedly consumed a commercial complementary food on the prior day, almost three quarters had consumed a commercially produced snack food product not specifically formulated for young children. One‐fifth of mothers reported having seen, read or heard a promotion for a commercially produced complementary food, while 85.4% reported having seen, read or heard a promotion for a commercially produced snack food product (Pries *et al.* 2016b).

In Phnom Penh, Cambodia, promotion of breastmilk substitutes was pervasive. Eighty‐six per cent of mothers reported observing a promotion for breastmilk substitutes. Consumption of breastmilk substitutes was also high, at 43.1% for children 0–5 months and 29.3% for children 6–23 months (Pries *et al.* 2016c). Among children 6–23 months of age, only 5.4% had consumed a commercially produced complementary food on the prior day, while 55.0% had consumed a commercially produced snack food product on the prior day. While over a quarter of mothers reported having observed a promotion for a commercially produced complementary food, almost all mothers (96.9%) had observed a promotion for a commercially produced snack food product (Pries *et al.* 2016d).

Feeley *et al*. report results from Dakar, Senegal, where breastmilk substitutes were given to 10.7% of infants <6 months of age and 20.2% of those 6–23 months of age. Of children 6–23 months of age, 50.5% had consumed a homemade complementary food, 49.1% had consumed a commercially produced complementary food and 58.7% ate a commercially produced snack food product on the previous day. Promotion of breastmilk substitutes and commercially produced complementary foods outside health facilities was common with 41.0% and 37.2% of mothers, respectively, having heard, seen or read product promotions since the birth of their youngest child. Promotion of commercially produced snack food products was more prevalent, with 93.5% of mothers having heard, seen or read such a promotion (Feeley *et al.* 2016).

In Tanzania, maternal exposure to commercial promotions for breastmilk substitutes and commercially produced complementary foods was low. Consumption of breastmilk substitutes was not prevalent, and only 3.1% of 6–23 month olds consumed commercially produced infant cereal on the day preceding the interview. Commercially produced snack food products were consumed by 23.1% of 6–23 month olds. Among infants less than 6 months of age, rates of exclusive breastfeeding were low (40.8%), and a high proportion of children less than 6 months of age (38.2%) received semi‐solid foods (Vitta *et al.* 2016).

**Figure 1:**
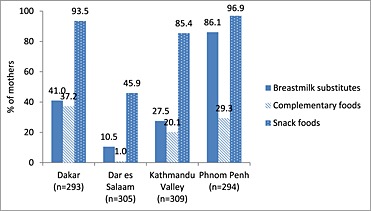
Percentage of mothers who observed a promotion for breastmilk substitutes, commercially produced complementary foods and snack foods since the birth of their child <24 months of age.

**Figure 2:**
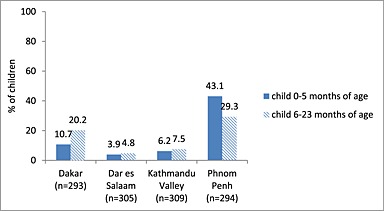
Percentage of children consuming breastmilk substitutes during the preceding day by age of child.

**Figure 3:**
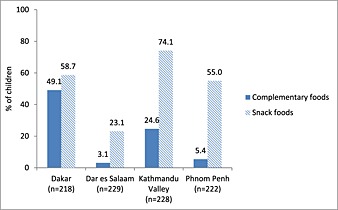
Percentage of children 6–23 months of age consuming commercially produced complementary foods and snack foods during the preceding day.

Rates of promotion and use of breastmilk substitutes and commercially produced complementary foods for the four sites are shown in Figs 1, 2, 3.

Food labels provide basic product information to product users on health, safety and nutrition and serve as a vehicle for food marketing, promotion and advertising (Canadian Food Inspection Agency 2011). The next article in this issue by Pereira *et al*. assessed labelling of breastmilk substitutes in comparison with a set of criteria based on the *Code* and subsequent relevant World Health Assembly Resolutions. Pereira's systematic assessment found that follow‐up formula and ‘growing‐up’ milks for older infants were frequently labelled similarly to infant formula products and a wide range of ages of introduction and descriptive names were found on the labels of these breastmilk substitutes across all four study sites (Pereira *et al.* 2016).

Sweet *et al*. assessed labels of commercially produced complementary foods compared with the intent of *the Code*. This assessment of labels from all four study sites indicated that labelling practices did not fully comply with international guidance and selected aspects of national legislation on the promotion of these foods. Inappropriate practices found to be prevalent included the lack of an appropriate age of introduction, lack of accurate and complete infant and young child feeding messages, recommended daily rations in excess of the daily requirements for breastfed children, and cross‐promotion between complementary foods and breastmilk substitutes produced by the same manufacturer (Sweet *et al.* 2016).

The final article in this supplement by Champeny *et al*. assessed advertising of products through promotions at the point of sale, (retail locations where products are sold). Researchers collected data on promotion of breastmilk substitutes and commercially produced complementary foods in retail outlets in all four study sites and found a wide range in the prevalence of point‐of‐sale promotions for both breastmilk substitutes and commercially produced complementary foods. Of a selection of stores selling infant and young child feeding products in each study site, just over a third of stores in Phnom Penh and Dakar had point‐of‐sale promotions for breastmilk substitutes, while they were observed in less than 10% of stores in Kathmandu Valley and Dar es Salaam. The study found that commercially produced complementary foods were promoted in half of the sampled stores in Dakar, but less than 10% of stores in Phnom Penh, Kathmandu Valley and Dar es Salaam. Point‐of‐sale promotions across all sites varied in content and form (Champeny *et al.* 2016).

The WHO is working to develop the World Health Assembly‐requested guidance on inappropriate promotion of foods for infants and young children and countries are working to strengthen policies and programmes to improve infant and young child feeding. The articles in this supplement include data on the levels of promotion and, for the first time, detailed information of reported intakes of commercially produced snack food products in four urban sites in Africa and Asia and will provide context for this effort.

Governments have an important role to play in promoting and protecting optimal infant and young child nutrition. To protect breastfeeding and ensure optimal complementary feeding practices, governments must monitor how breastmilk substitutes, complementary foods and other commercially produced foods are marketed and promoted. The International Code of Marketing of Breast‐milk Substitutes and relevant subsequent World Health Assembly resolutions address the promotion of breastmilk substitutes. However, the Code needs to be fully adopted into national regulations and must cover the range of breastmilk substitutes including follow‐up formulas and ‘growing‐up’ milks. Once in place, national regulations must be monitored and enforced. Given that the market for breastmilk substitutes was $45 billion worldwide in 2014 (Rollins *et al.* 2016), financial penalties for Code violations must be significant in order to compel manufacturer compliance. The global community must work together to prevent inappropriate marketing of breastmilk substitutes and to hold companies accountable for their practices.

The requested draft guidance recently issued in 2015 by the WHO on inappropriate promotion of foods for infants and young children provides greater clarity on inappropriate marketing of complementary foods (WHA 2016). Country governments will need to implement this guidance while taking into account existing legislation and policies. Consumption of unhealthy commercially produced snack foods also needs to be addressed. The surprisingly high rates of consumption of snack food products, typically of poor nutritional quality, among young children in these study sites indicate that nutrition policies and programmes in these sites should better facilitate informed decision‐making by mothers regarding which foods to feed their children and encourage replacement of unhealthy commercially produced snack food products with more nutritious, affordable foods.
